# Does osteoporosis increase complication risk in surgical fracture treatment? A protocol combining new endpoints for two prospective multicentre open cohort studies

**DOI:** 10.1186/1471-2474-11-256

**Published:** 2010-11-09

**Authors:** Sabine Goldhahn, Franz Kralinger, Daniel Rikli, Marta Marent, Jörg Goldhahn

**Affiliations:** 1 AO Clinical Investigation and Documentation, Stettbachstrasse 6, 8600 Duebendorf, Switzerland; 2 Clinic for Traumatology and Sports Medicine of the University Innsbruck, Austria; 3 Department for Traumatology of the University Hospital Basel, Switzerland; 4 Research Department, Schulthess Klinik Zurich, Lengghalde 2, Zurich, Switzerland

## Abstract

**Background:**

With an ever-increasing elderly population, orthopaedic surgeons are faced with treating a high number of fragility fractures. Biomechanical tests have demonstrated the potential role of osteoporosis in the increased risk of fracture fixation complications, yet this has not been sufficiently proven in clinical practice. Based on this knowledge, two clinical studies were designed to investigate the influence of local bone quality on the occurrence of complications in elderly patients with distal radius and proximal humerus fractures treated by open reduction and internal fixation.

**Methods/Design:**

The studies were planned using a prospective multicentre open cohort design and included patients between 50 and 90 years of age. Distal radius and proximal humerus fractures were treated with locking compression 2.4 mm and proximal humerus internal locking plates, respectively. Follow-up examinations were planned for 6 weeks, 3 and 12 months as well as a telephone interview at 6 months. The primary outcome focuses on the occurrence of at least one local bone quality related complication. Local bone quality is determined by measuring bone mineral density and bone mineral content at the contralateral radius. Primary complications are categorised according to predefined factors directly related to the bone/fracture or the implant/surgical technique. Secondary outcomes include the documentation of soft tissue/wound or general/systemic complications, clinical assessment of range of motion, and patient-rated evaluations of upper limb function and quality of life using both objective and subjective measures.

**Discussion:**

The prospective multicentre open cohort studies will determine the value of local bone quality as measured by bone mineral density and content, and compare the quality of local bone of patients who experience a complication (cases) following surgery with that of patients who do not (controls). These measurements are novel and objective alternatives to what is currently used.

**Trial registration numbers:**

Clinical Trials.gov NCT01144208 and NCT01143675

## Background

Osteoporosis leads to rarefaction of the intrinsic bone structure [1]. Thinning of the cortical shell in combination with less and thinner trabeculae [2] leads to inferior mechanical properties, e.g. lower compression force [3]. The subsequent increased incidence of low-energy fractures is well-known [4,5]. It is also known from epidemiological studies that permanent, untreated osteoporosis significantly increases the risk for another fracture [6] and furthermore, aggravates fracture fixation. The latter has been demonstrated in a number of biomechanical experiments that assessed various implant configurations (e.g. single screws, screw-plate constructs, dynamic hip screws) at different bone locations (e.g. proximal humerus, proximal femur or vertebra) under different loading modes (e.g. quasi-static, limited cyclic) [7]. Screw pull-out is significantly associated with bone mineral density (BMD) [8] or more precisely with the minimal contact area between implant and low density bone [9]. Consequently, fixation strength should be affected by osteoporosis and a higher risk for fixation failure in the form of screw pull-out or cut-through for example, might be expected.

Despite these biomechanical findings, very few clinical studies have reported an association between osteoporosis and an increased risk of fixation failure [10,11]. A recent systematic literature review also revealed that despite strong biomechanical evidence, an association could not be reproduced in clinical studies [7]. Furthermore, two reasons for the missing link were identified: 1) the lack of appropriate local osteoporosis diagnostics, and 2) the lack of uniform definitions for complications. The first aspect seems logical since current osteoporosis diagnostic tools were developed only to predict an overall fracture risk and not the local risk of fixation failure. Eckstein et al showed a substantial heterogeneity in BMD across different measurement sites and also found that local bone strength is best predicted by site-specific measurement [12]. Thiele et al showed that locally measured bone morphometric parameters like cortical bone mass, cortical thickness and bone density can explain more than 80% of the pull-out strength at the proximal femur [13]. Seebeck et al found an increase of up to 93 and 98% considering the adjacent cortical thickness and cancellous bone density measurements along single screws, respectively [8]. All these findings demonstrate the need for appropriate local bone density/morphometry assessment to evaluate a possible association with fixation failure.

Reports on complications occurring after surgical fracture treatment are rare and usually accompanied with inconsistency. This is the main reason for the absence of a universal classification system defining complications and thereby, a lack of clinical correlation between osteoporosis and the risk of fracture fixation failure. A recent systematic literature review of orthopaedic randomised controlled trials revealed that awareness and quality of reporting are low among orthopaedic surgeons [14]. Some authors report all complications without stratification for the possible relation to the intervention, whereas others either do not observe complications or simply do not report them. Systematic complication definitions including a characterisation of their possible relation to the tested interventions are a major prerequisite for studies focusing on bone quality and fixation.

The focus of this article is to describe a protocol that was designed to examine a hypothesised association between local bone quality and the risk of fixation failure. Local bone quality is measured using an up-to-date diagnostic tool for assessing local BMD and the risk of fixation failure is derived from a final independent assessment of the number of complications related to a defined set of categorised factors, i.e. local bone, fracture, implant or the surgical application. The protocol is applied in two specific clinical studies focusing on common fracture locations often associated with osteoporosis, the distal radius and proximal humerus.

## Methods/design

### Study design

Both studies are designed as prospective, multicentre open cohort studies with a nested case-control design. Open cohorts do not select subjects according to the type of exposure variable but enrol all members of the population and determine subsequent exposure status. In this case, exposure (i.e. local BMD level) has already occurred, but is not a criterion for subject enrolment. The major advantage of this type of approach as compared to a classical case control design is that in addition to comparing the BMD in cases and controls, we can also obtain an estimate of the risk of experiencing a complication in the general population.

The studies will be conducted at several participating clinics from Austria, Germany, Switzerland, Italy, China, Singapore and the US; recruitment will take place at 11 hospitals for the distal radius and 9 for the proximal humerus studies (Table 1).

**Table 1:** List of participating clinics

Clinic	Distal radius	Proximal humerus
**Austria**		
University Clinic for Trauma Surgery, Graz	x	
Medical University Innsbruck, Department of Trauma Surgery and Sport Traumatology, Innsbruck		x
		
**Germany**		
Charité Campus - Virchow Clinic, Centre for Musculoskeletal Surgery, Berlin	x	x
Evangelical Diaconal Friederikenstift Hospital, Clinic for Trauma and Reconstructive Surgery, Hannover	x	x
Clinic "rechts der Isar“ of the Technical University Munich, Trauma Surgery, Munich	x	x
		
**China**		
Department of Orthopaedics and Traumatology, Queen Mary Hospital, Hong Kong		x
		
**Italy**		
University Hospital Careggi, Hospital for Hand Surgery and Reconstructive Microsurgery, Florence	x	
		
**Singapore**		
Singapore General Hospital, Orthopaedic Trauma Services	x	
		
**Switzerland**		
University Hospital Basel, Traumatology Department, Basel	x	x
Cantonal Hospital Lucerne, Department for Trauma Surgery, Lucerne	x	x
Cantonal Hospital Winterthur, Surgical Clinic, Trauma and Joint Surgery, Winterthur	x	x
City Hospital Triemli, Surgical Clinic, Zurich	x	x
		
**USA**		
Massachusetts General Hospital, Harvard Medical School, Boston MA	x	

The clinical investigation plan was approved by the local ethics committees of all participating centers. Informed consent is obtained from each patient before data collection.

### Identification and recruitment of study participants

Patients between and including 50 and 90 years old are eligible for a study. The main inclusion criterion is a radiologically confirmed closed distal radius or proximal humerus fracture not older than 7 or 10 days, respectively (Table 2 and 3). For proximal humerus cases, only those patients with normal age-dependent function prior to operation, a monotrauma, and fracture displacement with a segment displacement of 0.5 cm or angulation > 45° [15] except dislocated fractures of the greater and lesser tuberosity will be recruited (Table 3).

**Table 2:** Eligibility criteria for the distal radius fracture group

Inclusion criteria (distal radius)
• Radiologically confirmed closed fracture (≤ 7 days) of the distal radius
• Primary fracture treatment with a volar LCP 2.4 mm
• Age > 50 and < 90 years
• Willing and able to give written informed consent to participate in the study
• Willing and able to participate in the study follow-ups according to the study protocol
• Willing and able to comply with the postoperative management program
• Able to understand and read country national language at an elementary level
**Exclusion criteria (distal radius)**
• Ulna fracture (except an associated fracture of the ulnar styloid process)*
• Open distal radius fracture
• Concomitant contralateral radius fracture
• Previous distal radius fracture on either side after the age of 25
• Time to operation > 7 days
• Polytrauma
• Regular systemic therapy with corticosteroids due to chronic disease
• Legal incompetence
• Patient received radio- or chemotherapy prior to, during or within the last year
• Currently diagnosed with active cancer
• Recent history of substance abuse (i.e. recreational drugs, alcohol)
• Prisoner
• Currently involved in a pharmaceutical clinical study**
• Has a disease process that would preclude accurate evaluation (e.g. neuromuscular or rheumatic disease, significant psychiatric or metabolic disorders)

* a) simple fractures of the ulna neck, b) multi-fragmentary fractures of the ulna neck, c) fractures of the ulna head and d) fractures of the ulna proximal to the neck are excluded, while radio-ulna dislocation (fracture of the styloid process) may be included.

** Simultaneous participation in another orthopaedic/surgical study with the same or another fracture will be approved by the principal clinical investigator

**Table 3:** Eligibility criteria for the proximal humerus fracture group

Inclusion criteria (proximal humerus)
• Radiologically confirmed closed fracture (≤ 10 days) of the proximal humerus
• All dislocated (according to modified Neer*) proximal humerus fractures except dislocated fractures of the greater and lesser tuberosity
• Primary fracture treatment with a PHILOS plate
• Age ≥ 50 and ≤ 90 years
• Normal (pre-trauma) function of both shoulders according to age
• Monotrauma
• Willing and able to give written informed consent to participate in the study
• Willing and able to participate in the study follow-ups according to the study protocol
• Willing and able to comply with the postoperative management program
• Able to understand and read country national language at an elementary level
**Exclusion criteria (proximal humerus)**
• Open proximal humerus fracture
• Concomitant contralateral proximal humerus fracture
• Previous proximal humerus fracture on either side after the age of 25
• Time to operation > 10 days
• Polytrauma
• Cuff arthropathy of the contralateral proximal humerus
• Associated nerve or vessel injury
• Regular systemic therapy with corticosteroids due to chronic disease
• Legal incompetence
• Patient received radio- or chemotherapy prior to, during or within the last year
• Currently diagnosed with active cancer
• Recent history of substance abuse (i.e. recreational drugs, alcohol)
• Prisoner
• Currently involved in a pharmaceutical clinical study**
• Has a disease process that would preclude accurate evaluation (e.g. neuromuscular or rheumatic disease, significant psychiatric or metabolic disorders)

* Segment displacement > 0.5 cm or angulated > 45° [15].

** Simultaneous participation in another orthopaedic/surgical study with the same or another fracture will be approved by the principal clinical investigator

Patients with open fractures, a concomitant contralateral fracture at the same location, or a previous fracture on either side after the age of 25 years are excluded. Furthermore, patients receiving regular systemic therapy with corticosteroids due to chronic disease are excluded. For the proximal humerus study, patients are excluded as well when they had cuff arthropathy of the contralateral side or associated nerve or vessel injuries (Table 3).

### Treatment

At the distal radius, a volar 2.4 mm locking compression plate (LCP) is used for stable internal fixation with a volar approach according to Henry et al [16]. A proximal humerus interlocking (PHILOS) plate is used for primary treatment of the upper limb fracture group. Both implants (Synthes GmbH, Oberdorf, Switzerland) are applied using standard recommended surgical procedures [17,18].

### Primary outcome measure

The primary aim of both studies is to evaluate if patients who experience bone/fracture or implant/surgery complications following surgical treatment with open reduction and a fixation plate (cases) have poorer bone quality (determined by local BMD) compared to control patients who do not experience a complication; hence, whether there is an association between poor bone mineral density and the risk of complication. Therefore, the main outcome involves documentation of the occurrence of at least one local bone quality related complication during 12 months of follow-up. The classification of complications complies with the previously defined system of AO Clinical Investigation and Documentation [19]. The qualifying events include those directly involving either the local bone and fracture or the implant and its surgical application. A detailed definition of these clinical and/or radiologically detectable events for the distal radius and proximal humerus are listed in Table 4 and 5, respectively.

**Table 4:** Detailed list of categorised definitions for bone/fracture and implant/surgery complications located at the distal radius

Bone/fracture	
(Secondary) Loss of reduction = secondary fragment dislocation = redisplacement = loss of radial/ulnar length = dorsal angle change = dorsal or palmar shortening	Any change in intra-/extraarticular angles, radial/ulnar length or secondary fragment dislocation compared to the immediate postoperative measurements
	
Malunion	Healed fracture in deformity (e.g. varus/valgus, rotational malunion) as a consequence of loss of reduction
Malunion = residual extraarticular deformity = residual articular gap/step-off	Inadequate/insufficient anatomical reduction as a consequence of significant change of the postoperative position. Bone unites in abnormal position and/or alignment
	
Fracture impaction	Articular impaction of ≥ 1 mm
	
Delayed healing	Insufficient signs of healing (> 3 months)
	
Nonunion	Indiscernible signs of healing (> 6 months)
Refracture = secondary fracture	Fracture occurs at the same radial site with a load level otherwise tolerated by normal bone after the bone has solidly bridged
	
Functional deficit	As assessed with the functional scores of DASH, PRWE, ROM and grip strength

**Implant/surgery**	

Primary malpositioning of plate	Failure to fix each fragment and/or failure to implant plate according to technical guide [17] (including anatomical reduction and plate positioning) during operation
	
Primary malpositioning of screw	Screws placed into the radiocarpal or radioulnar joint as seen on oblique X-rays or incorrect screw size chosen during operation
	
Secondary screw perforation = secondary screw loosening = screw back-out	Screws displaced into the radiocarpal or radioulnar joint as seen on oblique X-rays or incorrect screw size chosen (no initial perforation)
	
Implant (plate and screw) loosening	Multiple proximal or distal screw loosening leading to firstly, relative movement between one main fragment and the plate and secondly, pull-out of the implant
	
Plate/screw failure/breakage	Plate/screw breakage
	
Radiolucency around screw	Appearance of screw on X-ray without screw loosening

DASH = Disabilities of the Arm, Shoulder and Hand questionnaire PRWE = Patient-Rated Wrist Evaluation questionnaire ROM = Range Of Motion

**Table 5:** Detailed list of categorised definitions for bone/fracture and implant/surgery complications located at the proximal humerus

Bone/fracture	
(Secondary) Loss of reduction = secondary fragment displacement = redisplacement	Significant change of head position against shaft or fragment dislocation compared to the immediate postoperative position
= secondary dislocation of greater tuberosity	Relative change of greater tuberosity compared to the immediate postoperative position
	
Malunion	Fracture healed with deformity and bone unites in abnormal position and/or alignment (e.g. varus/valgus, rotational malunion)
	1. as a consequence of loss of reduction
	2. as a consequence of inadequate/insufficient anatomical reduction as observed on postoperative X-rays.
	
Delayed healing	Insufficient signs of bridging/continuous fracture line still visible up to 6 months
Nonunion	Indiscernible signs of bridging/continuous fracture line still visible after 6 months
	
Head impaction = secondary fracture impaction	Secondary impaction/sintering of the head fragment with consecutive intraarticular screw penetration due to mechanical failure (not due to avascular collapse of the head fragment) compared to immediate postoperative position
	
Head necrosis	In situ death of bone within the humeral head due to disruption of blood supply, leading to partial or complete collapse of the head
	
Refracture = secondary fracture at the same site	After fracture is healed: refracture occurs at the same humeral site with a load level otherwise tolerated by normal bone after the bone has solidly bridged
	
Functional deficit	As assessed with the functional scores of DASH, SPADI, ROM and Constant-Murley

**Implant/surgery**	

Primary malpositioning of plate	Failure to fix each fragment and/or failure to implant plate according to technical guide [18] (including anatomical reduction and plate positioning i.e. at least 8 mm distal to the upper end of the greater tubercle) during operation
	
Primary malpositioning of screw	Screw perforation into glenohumeral joint during operation
	
Secondary screw loosening: back-out	Relative change outwards of screw position in relation to postoperative position
	
Secondary screw loosening: perforation	Perforation of one screw through the cortex of the head (no initial perforation)
	
Implant loosening = Proximal screw and plate pull out = Distal screw and plate pull out	Multiple proximal or distal screw loosening leading to firstly, relative movement between one main fragment and the plate and secondly, pull-out of the implant
	
Implant failure/breakage	Plate/screw breakage

DASH = Disabilities of the Arm, Shoulder and Hand questionnaire SPADI = Shoulder Pain And Disability Index ROM = Range Of Motion

All radiographs will be initially assessed for wrist alignment measurements and complication events by an independent radiologist. A final classification of all complications will be performed after the end of the 12-month follow-up period by an independent study review board using anonymised data and radiographs.

### Secondary outcome measures

#### Documentation of additional complications

The occurrence of any complication other than specific bone/fracture and implant/surgery events during the 12-month follow-up period will also be assessed for both studies. "Any" complication comprises soft tissue/wound and systemic/general complications.

Superficial and deep wound infections, nerve irritation or injury, and local pain are considered as "soft tissue/wound" complications. Reflex sympathetic dystrophy, carpal tunnel syndrome, tendon irritation or rupture, ganglion, and neurological symptoms (e.g. dysesthesia) are additionally included under the definition of a soft tissue complication specifically occurring at the distal radius. For the proximal humerus, additional soft tissue complications include impingement, loss of range of motion, stiffness and rotator cuff lesions.

Systemic/general complications comprise infections localised at any site other than the fractured region, sepsis, thrombosis, embolism, and death.

#### Determination of bone mineral density (BMD)

The standard diagnostic method for classifying osteoporosis is the measurement of BMD at specific body sites, namely the hip, vertebra or radius. The most commonly used tools for BMD measurement are dual energy X-ray absorptiometry (DXA), quantitative computed tomography (QCT), and quantitative ultrasound [20,21].

Despite the well-known problem of comparing measurement devices, target regions of interest (ROI), and reference measurements of different populations, the World Health Organization defines DXA as the method most highly developed technically and most thoroughly validated biologically. It is regarded as the 'gold standard' with which the performance characteristics of less well-established techniques can be compared [22]. Therefore, DXA will be used for the nominated studies.

Although BMD is most frequently determined at the lumbar spine, total hip and proximal femur, a peripheral DXA method focused at the distal radius will be used. This technique has high precision accompanied by low radiation exposure levels [21]. Sievanen et al evaluated the in vivo day-to-day precision of DXA for seven anatomic sites in the upper left and right extremities of ten subjects. The determined precision values of BMD for the proximal humerus and distal radius were 0.8% and 0.7%, respectively [23].

For both studies, local bone status will be assessed by determining the BMD of the contralateral radius. Absolute values will be documented as well as the T-score according to the DXA manufacturer's reference collective, e.g. -1.5 standard deviations (STD). Comparisons of BMD measurements obtained from the left and right humeri revealed higher correlations between the distal (r = 0.95) and proximal humerus (r = 0.86) of both arms when compared to the proximal and distal humerus (r = 0.63) of the same arm [24]. These data suggest that the contralateral side is an appropriate estimate of the bone quality of a fractured side.

#### Functional and quality of life assessments

Regular physical exam of the wrist or shoulder involves the determination of range of motion [25].

Patient self-evaluation of their upper limb function and health related quality of life status will be assessed using the Disabilities of the Arm, Shoulder and Hand (DASH) [26] and EuroQol 5 [27] questionnaires, respectively. The DASH is a 30-item survey which will be used in its original or translated validated versions, and allows the patient to rate and describe their upper limb disability during the last week as well as monitor changes in symptoms and function over time. For the health related quality of life assessment, an adapted version of the EuroQoL 5 will be used. The original 20 cm visual analogue scale with endpoints labelled "worst imaginable health state" and "best imaginable health state" anchored at 0 and 100 respectively, has been changed to an 11-point visual numeric scale ranging from 0 to 10. This alternate response format has already been used in clinical studies and is more suitable for the telephone interview. Because the baseline questionnaire will be filled out by the patient after the accident and operation, the question "please indicate how you feel today" will be modified to "please indicate how you felt before the operation" in order to obtain a pre-injury baseline value.

#### Specific secondary outcome assessments for the distal radius

Grip strength will be evaluated using a Jamar dynamometer (Sammons Preston Roylan, Bolingbrook IL, USA). The average value of three successive measurements will be calculated as a percentage of the contralateral, healthy side [28]. An impairment of muscle power is defined as a clearly lower average value for the injured leading hand compared to the opposite hand. If the injury involves the non-leading hand, the average value must be 50% lower than that for the opposite hand [29].

The Patient-Rated Wrist Evaluation questionnaire (PRWE) is a 15-item survey which allows patients to self-assess their levels of pain and disability in activities of daily living. It will be used as published [30] or in a translated validated version according to the guidelines of Beaton et al [31].

In a subset of patients, the bone volume/trabecular volume (BV/TV) ratio will be measured using high resolution peripheral quantitative computed tomography (Xtreme CT) (Scanco Medical AG, Brüttisellen, Switzerland) and compared with DXA measurements.

For the measurement of distal radius BMD, the XTreme CT will be calibrated with the aid of a reference phantom [European Forearm Phantom (EFP-02-45; QRM, Moehrendorf, Germany)] consisting of a series of various hydroxyapatite densities.

#### Specific secondary outcome assessments for the proximal humerus

The Constant-Murley score is a 100-point functional shoulder assessment tool in which higher scores reflect increased function. It combines four separate subscales focusing on subjective pain, function, objective clinician assessment of range of motion and strength. The score will be used as originally published [32,33].

The Shoulder Pain And Disability Index (SPADI) is a self-administered questionnaire that assesses the severity of pain and the degree of difficulty with various activities of daily living that require upper extremity use. It will be used as published or in its validated version [34]. As no Chinese (Cantonese) validated version of the SPADI exists, formal translation according to established guidelines [31,35,36] has been performed. The Chinese version has been approved by the developer and will be pretested in the present study in Hong Kong.

A CT based method for the measurement of BMD at the proximal humerus will be validated, and subsequently used to preoperatively determine BMD for the contralateral limb [37]. Briefly, simultaneous CT imaging of the contralateral proximal humerus with a European Forearm Phantom (EFP-02-45; QRM, Moehrendorf, Germany) is performed; the phantom is placed on the midthorax of the patient. After acquisition, the measured CT units (Hounsfield Units [HU]) of the user-defined ROI can then be quantified from the EFP-based calibration curve, resulting in definitive BMD values. This CT assessment of proximal humerus BMD will then be compared to the DXA measurements used to quantify BMD of the distal radius.

**Figure 1:**
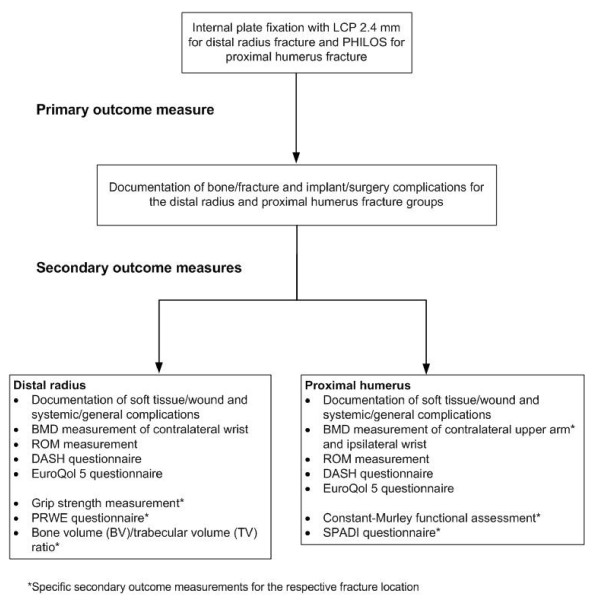
**Flow chart of the study protocol**.

A summary of all outcome measures assessed during each study is presented in Figure 1.

### Timing of outcome assessments

For both studies, patients will be followed from the day of initial treatment to one year thereafter. The follow-up includes initial hospitalisation (baseline) and follow-up visits at 6 weeks, 3 months and 12 months following surgery. At these follow-ups all outcomes except radiological data will be measured in the hospitals. Any unplanned follow-up visits resulting from the occurrence of a complication during the 12-month period will also be documented. Additional telephone interviews will take place at the 6-month postoperative period to obtain the patient self-rating PRWE, SPADI, and EuroQol5 questionnaire evaluations.

### Safety monitoring

Due to the investigational nature of the study to evaluate the association of local BMD with the occurrence of specific types of treatment complications, the entire reporting process of complications/adverse events (AE) is adapted such that the investigators are obligated to report all types of events, independent of being classified as a complication or an AE.

A complication is defined as any occurrence or deviation from the normal treatment or outcome, regardless of whether it has any relationship with the treatment or product under investigation.

All complications occurring at any time during the patient's postoperative follow-up period will be reported by the subject or observed by the investigator and documented in a separate complication form, whether or not the investigator concludes the event to be related to the treatment. The form will include the following information:

▪ type and date of event

▪ study name and the patient identification and treatment number

▪ investigator name and phone number

▪ name of the suspect medical product and the day of implantation

▪ a short description of the complication

▪ event seriousness and relation to study participation

▪ most likely causative factor

▪ event treatment

▪ event outcome and the relation (if any) of the event to the treatment procedure

### Sample size and statistical analysis

The primary objective of both studies is to assess BMD as a risk factor for the development of treatment complications. This objective will be analysed based on a nested case-control design. Using this statistical approach, the outcome variable is the occurrence of any complication and the exposure variable is the BMD measurement at the contralateral side. Cases include subjects reported with any bone and fracture related complication during the study duration of 12 months. Controls are subjects without any recorded complication. Thus, the main conceptual approach in this analysis is the difference in population BMD means between the cases and controls.

A two-way superiority hypothesis that the BMD is lower in cases compared to controls will be tested. The appropriate approach is to test the null hypothesis which states that the BMD is equal in cases and controls, i.e. H0: μcontrols = μcases or H0: μcontrols - μcases = 0, where controls and cases are the means of the two independent normal distributions. The alternative hypothesis is that BMD is different between the cases and the controls, i.e. H1: μcontrols ≠ μcases or H1: μcontrols - μcases ≠ 0. Rejection of the null hypothesis leads to the acceptance of the alternative hypothesis. The expected direction is that BMD is lower among the cases, which suggests the association between low BMD and the occurrence of complications. The expected ratio of cases versus controls is 15:85 for distal radius fractures and 30:70 for the proximal humerus. A standard deviation of 0.05 g/cm^2 ^for radius BMD measurements has been estimated [38]. With an expected BMD of 0.4 g/cm^2 ^at the radius and 200 subjects, the distal radius study has 85% power to detect a difference of -0.03 g/cm^2 ^in BMD at the wrist between the cases and the controls (two-way test). This corresponds to a Cohen effect size of 0.6, which indicates a medium clinical difference. With a power of 85%, a significance level of 0.05 (two-sided), an additional 10% in the number of subjects to compensate for any loss of power due to an adjustment for imbalance between the groups, and an estimated 10% loss to follow-up at the 1-year clinical examination, the distal radius and proximal humerus studies require 244 and 148 patients, respectively. Our sample size estimates are based on the following series of assumptions: 1) the proportion of cases in the patient population (i.e. % subjects with any complication); 2) the standard deviation of BMD at distal radius; and 3) the difference of μ_controls _- μ_cases_. Sample size estimates were performed using the Study East Version 4.0.1 (Cytel Statistical Software, Cambridge MA, USA).

## Discussion

Although an association between local bone quality and implant anchorage has been proven in various in vitro settings, clinical evidence is still lacking [7]. Case reports as well as clinical experience suggest that fractures in osteoporotic patients are at higher risk of implant complication. Quantification of the problem is required in order to a) determine the size of the problem, b) provide numbers and characteristics for implant development, c) establish a basis for treatment decision-making, and d) support teaching in the field of geriatric fracture care. The proof of the hypothesised association is only possible in a controlled setting. Therefore, our study approach focuses on two major osteoporosis related indications. Distal radius fractures are regarded as sentinel fractures. Previous studies show high correlations between the degree of osteoporosis and radiological healing criteria [39,40], but surgical treatment with locked plates may have a positive impact on this correlation. For proximal humerus fractures, the use of locked plates does not seem to prevent all complications; rates of up to 40% are reported in the literature [41,42]. In this context, it is interesting to evaluate whether local bone quality is a significant contributing factor.

Although the study goals and methodology are novel, a few assumptions need to be made. Since local bone quality cannot be measured directly at the fracture site, remote sites have to be evaluated. Previous work has shown that the contralateral site serves as a better surrogate than ipsilateral locations in other areas [24]. Yet conditions such as post-stroke hemiparesis have an influence on these elements. Therefore, in the case of a proximal humerus fracture, BMD will be measured at the ipsilateral distal radius as well as at the contralateral proximal humerus. In the case of a distal radius fracture, only the contralateral side can be utilised to estimate BMD at the fracture site.

The latest concept of complication assessment is utilised for this study [19]. The main feature is that it distinguishes between local and general complications, with the former divided into further subcategories of bone/fracture, implant/surgery, and soft tissue associated complications. This should enable the investigator to establish causality. Overlapping between the categories may influence the assessment. The new feature of centralised radiograph and complication assessment will greatly help to improve data quality and may minimise investigator bias in the assessment of cases and controls.

Despite significant efforts to unify surgical care in both treatment arms, many confounding variables may influence the hypothesised association. Differences in the surgical approach, surgical skills, hospital infrastructure and postoperative rehabilitation may contribute to variability in the final result, thus influencing and possibly masking a bone related effect. Nevertheless, the study protocol adopts a real-world approach and therefore, represents current clinical practice rather than a theoretical framework.

## Competing interests

The authors declare that they have no competing interests.

## Authors' contributions

All authors have read and approved the manuscript and believe its content represents honest work. All authors (SG, FK, DR, MM and JG) have made substantial contributions to the design of the presented study. SG and JG have written the first manuscript, and DR, FK and MM have reviewed the manuscript.

## Pre-publication history

The pre-publication history for this paper can be accessed here:

http://www.biomedcentral.com/1471-2474/11/256/prepub
